# Evaluation of micro-dosing fertilizer application on sorghum (Sorghum bicholor L) production at Wag-Lasta Areas of Amhara Region, Ethiopia

**DOI:** 10.1038/s41598-020-63851-6

**Published:** 2020-04-23

**Authors:** Workat Sebnie, Merse Mengesha, Gebrehana Girmay, Tesfaye Feyisa, Belaynesh Asgedom, Gashaw Beza, Dereje Dejene

**Affiliations:** 1 Sekota Dry-land Agricultural Research, Center, P.O. Box 62, Sekota, Ethiopia; 2 Amhara Agricultural Research Institute P.O.Box 527, Bahir Dar, Ethiopia; 3 Wolkitie University P.O.Box 07, Wolkitie, Ethiopia

**Keywords:** Plant sciences, Ecology, Environmental sciences

## Abstract

Soil fertility management through inorganic fertilizer application in areas with moisture shortage needs due care. The fertilizer application fashion should vary from areas with ample moisture so that the plants can easily access and efficiently use it. Therefore, field experiment was conducted in 2014 and 2015 during the main cropping season under rain-fed condition to evaluate the effect of micro-dose application of N and P fertilizers on sorghum yield at Aybra and Shumshiha sites of Wag-Lasta areas in Amhara Region, Ethiopia where moisture shortage is acute. The treatments were comprised of a factorial combination of three levels of NP i.e. 1), 25% of the recommended NP = 10.25 kg N + 11.5 kg P_2_O_5_ ha^−1^_,_ 2), 50% of the recommended NP = 20.5 kg N + 23 kg P_2_O_5_ ha^−1^ and 3), 75% of the recommended NP = 30.75 kg N + 34.75 kg P_2_O_5_ ha^−1^ and three N application times plus control (without fertilizer) and recommended NP (41 kg N ha^−1^ and 46 kg P_2_O_5_ ha^−1^). The experiment was laid down in a randomized complete block design with three replications. The fertilizers were applied to the spot where the seed was planted except for the recommended NP which was drilled to the planting rows right before planting. Phosphorus was applied at planting while nitrogen was applied in split as per the treatment setup. All soil and agronomic data were collected and analyzed following the standard procedures. The analysis of variance revealed that application of 30.75 kg N ha^−1^ and 34.5 kg P_2_O_5_ ha^−1^ (N applied 1/3 at sowing, 1/3 at emergence and 1/3 at 45 days after emergence) increased the grain yield by 122% over the control and 28.4% over the recommended NP and saves 25% of the recommended fertilizer at Aybra. While at Shumshiha-Lasta Lalibela, application of 20.5 kg N ha^−1^ and 23 kg P_2_O_5_ ha^−1^ (N applied 1/3 at sowing, 1/3 at emergence and 1/3 at 45 days after emergence) increased the grain yield by 174% over the control and 15% over the recommended NP and saves 50% of the recommended fertilizer. Therefore, micro-dosing application of 30.75 kg N ha^−1^ and 34.5 kg P_2_O_5_ ha^−1^ for Aybra-Sekota and of 20.5 kg N ha^−1^ and 23 kg P_2_O_5_ ha^−1^ for Shumshiha-Lasta Lalibela (N applied in three splits) are recommended for sorghum production.

## Introduction

Low levels of soil fertility because of land degradation and nutrient depletion has been a critical challenge to agricultural production in Ethiopia. On cultivated land, there is a persistent decrease in soil quality coming about because of diminished fallows and imperfect utilization of inputs. Continuous cultivation coupled with nutrient depletion, poor crop residue management, and reduced crop rotation resulted in poor soil fertility^1^. Most soils in the semi-arid areas of northeastern Ethiopia are intensively depleted of the plant nutrients especially total nitrogen, available phosphorus and organic carbon are characterized by low category for this reason they leading to substantial decline in crop productivity^2^.

Sorghum is one of the important food crops in Ethiopia that comprises 17% of the total cereal production in the country. It accounts for more than 655671.97 ha total cultivated area in Amhara Region^3^. But, the average yield of sorghum per unit area is not more than 1.2 t ha^−1^^3^. Low, erratic, unevenly distributed rainfall and poor soil fertility are some of the causes of low crop productivity including sorghum in Ethiopia^4^.

Inorganic fertilizer is critically important to increase crop yield^5,6^. Suggested that fertilizer rates must be expanded to fulfill the regularly expanding need for nourishment. Micro-dosing refers to the application of small quantities of fertilizer at planting or as top dressing about three to four weeks after emergence. Micro-dosing fertilizer increases fertilizer use efficiency and improves yields, while minimizing input and investment cost. This is an effective method to apply fertilizer, because the fertilizer is applied 5 cm adjacent to the seeds, thereby ensuring a high rate of uptake. Micro-dosing of fertilizers was found to increase yields by 44% to 120% and farmers’ income by 52% to 134% compared to traditional application methods (basal application and top dressing)^7^. Similar research findings in Niger show that micro-dose method increased yield with low cost and efficient^8^. Likewise^9^, showed that farmers could boost their yields by 50% by applying about 9 kg of nitrogen per hectare compared to no application in Zimbabwe. In addition, rational use of fertilizer plays its own role to mitigate climate change^10^. Similarly, micro-dosing fertilizer application could increase the fertilizer use efficiency for the fertilizer is applied to the root zone of the plant and can easily be taken by the plant roots. However, there is no information on fertilizer application in micro-dosing in the study area. Therefore, this research was designed to evaluate micro-dosing fertilizer application techniques (N and P) on sorghum yields at moisture stressed areas of Sekota and Lasta Laibela districts of Amhara region.

## Materials and method

### Description of the study areas

**Figure 1:**
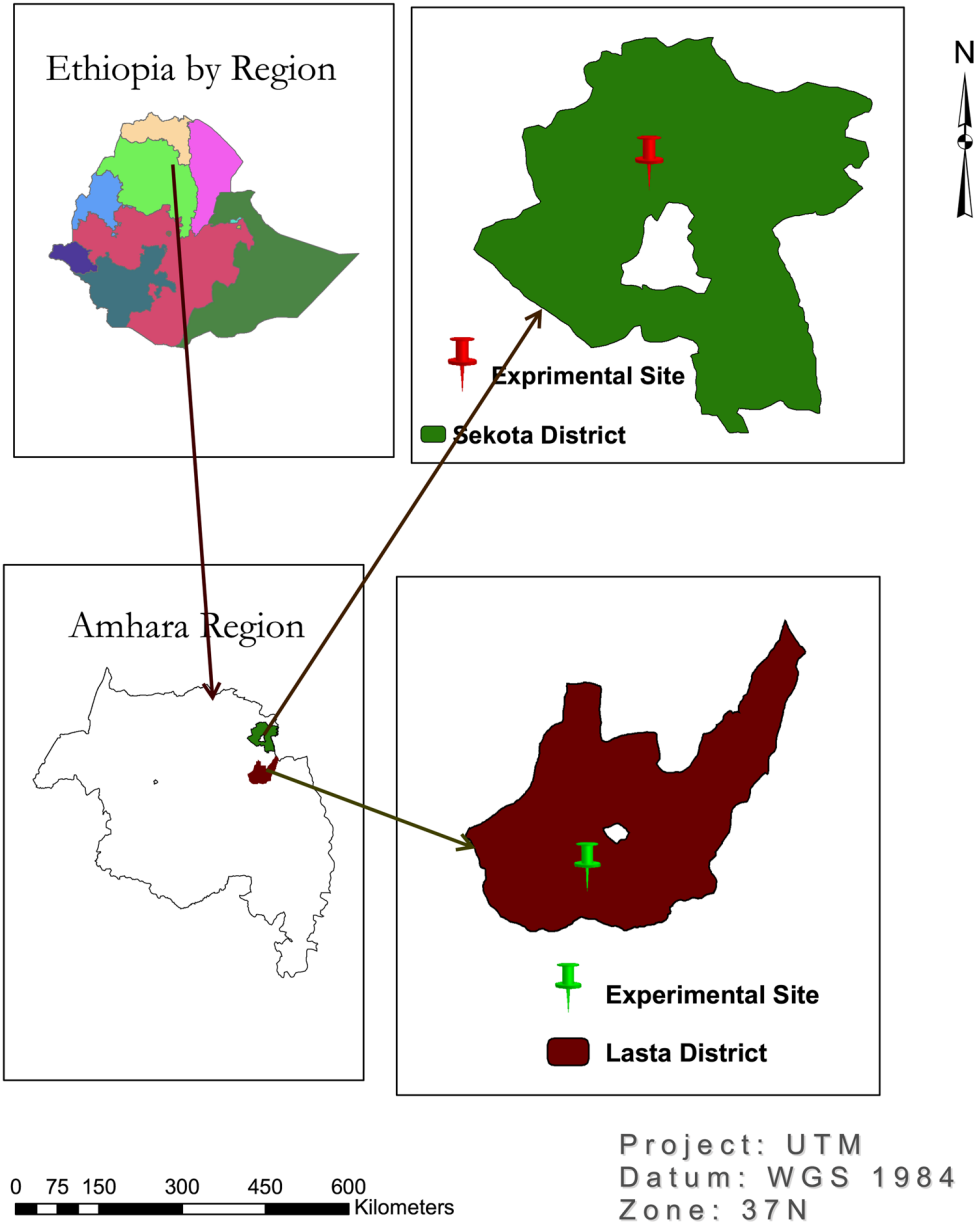
Location map of study area.

The study was conducted for two years in 2014 and 2015 at Aybra-Sekota Shimshiha-Lasta Lalibela areas of Amhara Region, Ethiopia respectively (Fig. 1). The location of the study area was found within an altitude ranging from 1921 to1947 m.a.s.l. The study areas are characterized by small (450 mm to700 mm) with erratic rainfall.

### Experimental design

The experiment was comprised of three NP rates (i.e. 1) 25% of the recommended NP 10.25 kg N ha^−1^ + 11.5 kg P_2_O_5_ ha^−1^, 2) 50% of the recommended NP = 20.5 kg N ha^−1^ + 23 kg P_2_O_5_ ha^−1^ and 3) 75% of the recommended NP = 30.75 kg N ha^−1^ + 34.5 kg P_2_O_5_ ha^−1^) factorially combined with three nitrogen application times (i.e. 1. Two splits: half at sowing and half at emergence, 2. Three splits: one third at sowing, One third at emergence and one third at 45 days after emergence, 3. Two splits: Half at sowing and half at 45 days after emergence) arranged in a completely randomized block design (RCBD) with three replications. In addition, control (without any nutrient) and recommended NP (100 kg ha^−1^ DAP and 50 kg ha^−1^ urea) were included to the treatments and made a total of 11 treatments. The recommended NP was applied to the planting rows by drilling (all P once at planting with half of N and the other half of N 45 days after planting). Immediately at planting. Whereas the other fertilizer treatments were applied in a micro-dosing to the spots where the seeds were planted (to the root zone 5 cm soil depth) as per the treatment setup and slightly covered by soil to avoid a direct contact of the seeds with the fertilizers. Urea and DAP were used as a source of nitrogen and phosphorus respectively. Urea was applied in split as per the treatments while DAP was applied once at planting. Each plot was having an area of 15m^2^ (3.75 m by 4 m). The space between blocks was 1 m and between plots was 0.5 m. The space between plants and rows was 15 cm and 75 cm; respectively. Misker sorghum variety was used as a test crop. Tie-ridge with 2 meter interval was uniformly applied for all treatments for moisture conservation. Similarly, cultivation and weeding were done uniformly for all treatments.

### Data collected

The grain yield was measured after harvesting the above-ground biomass at full physiological maturity from the central rows of the plot excluding the border rows, sun drying, measuring the above-ground biomass (in kg per plot) and then threshing and separating the grains from the straws. The grain yield per plot was reported in kg and was converted to kilograms per hectare. The above-ground biomass yield in kg per plot was also converted to kg per ha.

### Data analysis

Collected datas were subjected to statistical analysis using SAS statistical software version 9.0 and significant treatment effects were compared using the Fisher’s Least Significant Differences test at 5% level of significance.

### Soil sampling and analysis

A composite soil sample was collected from 0–20 cm before sowing, air-dried and passed through 2 mm sieve to determine pH, ECe and passed through 0.5 mm for total nitrogen and organic carbon. The soil parameters were determined following the standard laboratory procedures. Soil pH was determined in H_2_O using 1:2.5 soils to solution ratio using a combined glass electrode pH meter^11^. Soil organic carbon was determined following the wet digestion method as described by^12^ while percent organic matter of the soils was determined by multiplying the percent organic carbon value by 1.724. Total N was analyzed by the Kjeldahl digestion and distillation procedure^13^.

### Partial budget analysis

The cost benefits and marginal rate of returns of the treatments were analyzed based on the technique described by^14^. To estimate the economic parameters, the grain of sorghum was valued based on average market price collected from the local markets during the two consecutive production years. The average cost of Urea and DAP fertilizer and labor cost for each fertilizer application were collected from the two districts. The average yield was adjusted downward by 10% from the exact yield to reflect the difference between the experimental yield and yield of farmers. MRR (%) was calculated as changes in net benefit divided by changes in cost.

### Ethics approval and consent to participate

It is to declare that we have all the ethical approval and consent from our Research Institute to participate in research paper writing and submission to any relevant journal.

## Results and discussion

The soil pH of the surface soil for Aybra-Sekota and Shumshiha-Lasta Lalibela was 6.3 and 6.4 respectively (Table 1). Based on^15^, the reaction of the study areas is within slightly acidic class. The organic matter content of the soil was 1.0% for Aybra and 1.2% for Shumshiha-Lasta Lalibela. According to^16^ rating of organic matter it was rated as low. Similarly, the total nitrogen content was 0.01% for Aybra-Sekota and 0.02% for Shumshiha-Lasta Lalibela; which is extremely low according to rating given by^15^.

**Table 1:** Selected soil properties for the study areas.

Location	pH	EC	%OM	%TN
Aybra-Sekota	6.3	0.16	1.17	0.01
Shumshiha-Lasta Lalibella	6.4	0.13	1.00	0.02

## Effect of nitrogen and phosphorus fertilization on grain and biomass yields

### Effects of nitrogen and phosphorus on sorghum grain yield

The maximum grain yield (2476.4 kg ha^−1^) was obtained from 34.5 kg P_2_O_5_ and 30.75 kg N ha^−1^, (N applied in three splits) while the minimum grain yield (1114.8 kg ha^−1^) was obtained from the control (without fertilizer) at Aybra-Sekota. Micro-dosing application increased the yield by 122% over the control and by 28.4% over the recommended NP (Table 2) at Aybra-Sekota. Similarly, the maximum grain yield (2476.6 kg ha^−1^) was obtained from 20.5 kg N ha^−1^ and 23 kg P_2_O_5_ ha^−1^ (Nitrogen applied in three splits) whereas the minimum yield (1023 kg ha^−1^) was obtained from the control (without fertilizer) at Shumshiha-Lasta Lalibela (Table 2). In addition to the yield advantages over the recommended NP, micro-dose application saved 25% fertilizer at Aybra and 50% fertilizer amount at Shumshiha-Lasta Laibela. The saved fertilizer amounts can be used for additional sorghum production on 1/3 ha at Aybra-Sekota and on one ha at Shumshiha in a micro-dose application. The result is in line with the findings of ^7^ and^17^ who reported that sorghum yield increment ranging between 44 to 120% compared to the control using micro-dose application. Similarly^18^, reported that application of micro-dose of 10 kg nitrogen ha^−1^ increased the yield by 30–100%. Three times split application of nitrogen with micro-dosing 1/3 at sowing, 1/3 at emergence and 1/3 at 45 days after emergence was more efficient than the other application methods.

**Table 2:** Effect of nitrogen and phosphorus fertilization on biomass and grain yield.

P_2_O_5_kg ha^−1^	N kg ha^−1^	Aybra-Sekota						Shumshiha-Lasta Lalibela					
		Grain yield kg ha^−1^			Biomass yield kg ha^-1^			Grain yield kg ha^-1^			Biomass yield kg ha^-1^		
		Year-1	Year-2	Average	Year-1	Year-2	Average	Year-1	Year-2	Average	Year-1	Year-2	Average
0	0	1324.1	905.6	1114.8	4548.1	3944.4	4246.3	1573.9	472.2	1023.1	5933.5	1944.4	3939
11.5	10.25 SE	1116.7	1911.1	1513.9	7225.3	6111.1	6443.1	1977.8	555.6	1266.7	7186.7	1333.3	4260
11.5	10.25 SE45	1738.1	1662.2	1700.1	5572.5	5611.1	5591.8	1952.5	616.7	1284.6	7248.9	2777.8	5013
11.5	10.25 S45	1535.0	1861.1	1698.0	6486.5	5555.6	6021.0	1830.0	1026.6	1427.3	7775.2	3055.6	5415
23	20.5SE	1760.0	2277.8	2018.9	5920.0	5833.3	5876.7	2833.6	1311.1	2072.4	10767	3222.2	6995
23	20.5 SE45	2143.9	1777.8	1960.8	6216.9	5740.7	5978.8	2715.6	2900.0	2807.8	9863.1	4722.2	7293
23	20.5 S45	1960.3	2066.7	2013.5	7672.2	3888.9	5780.6	2101.9	2002.3	2045.1	8622.8	2500.0	5561
34.5	30.75 SE	1540.8	2738.9	2139.9	6090.0	5000.0	5545.0	2329.6	2577.8	2453.7	9581.9	5185.2	7384
34.5	30.75 SE45	2158.7	2794.4	2476.6	7219.4	5666.7	6668.2	2148.7	1700.0	1924.4	8628.1	5277.8	6953
34.5	30.75 S45	1816.5	2272.2	2044.4	5767.5	4111.1	4939.3	2017.8	2250.0	2133.9	8388.3	3888.9	6139
46	41	1952.2	1905.6	1928.9	6193.5	4777.8	5485.6	2280.6	2594.4	2437.5	8804.1	4444.4	6624
CV (%)		12.89	15.23	21.47	9.54	13.22	17.73	7.73	11.92	24.74	10.3	14.87	27.82
LSD (0.05)		380.25	535.52	474.2	1012.5	1144.6	1167.2	288.47	362.02	572.01	1464.3	878.06	3298.6

SE stands for urea application at sowing and at emergence; SE45 stands for urea application at sowing, at emergence and at 45 days after emergence, S45 stands for urea application at sowing and at 45 days after emergence.

### Effects of nitrogen and phosphorus on sorghum biomass yield

At Aybra-Sekota significantly higher biomass yield (6668.2 kg ha^−1^) was obtained from 34.5 kg P_2_O_5_ and 30.75 kg N ha^−1^, (N applied in three splits) while the minimum biomass yield (4246.3 kg ha^−1^) was obtained from the control (Table 2). At Shumshiha-Lasta Lalibela the maximum biomass yield (7293 kg ha^−1^) were obtained from 23 kg P_2_O_5_ ad 20.5 kg N ha-1 (Table 2). This implies that micro-dose application increased the biomass yield by 57% over the control and by 21.6% over the recommended NP at Aybra-Sekota and by 85% over the control and 10% over the recommended NP at Shumshiha-Lasta Lalibela. The results are in line with the findings of [7, 17, and 18] who reported increased sorghum biomass due to micro-dose application.

### Partial budget analysis

Partial budget analysis of Aybra-Sekota shows that application of 34.5 kg P_2_O_5_ and 30.75 kg N ha^−1^ (N applied in three splits) had the highest net benefit (14051.07 ETB ha^−1^) with MMR of 518.95% (Table 3). Whereas the partial budget analysis for Shumshiha-Lasta Lalibela shows that application of 20.5 kg N and 23 kg P_2_O_5_ ha^−1^ (N applied in three splits) resulted in the highest net benefit (17844.01 ETB ha^−1^) (Table 4) with MRR of 14942.3%.

**Table 3:** Partial budget analysis for Aybra-Sekota.

P_2_O_5_ kg ha^−1^	N kg ha^−1^	Unadjusted yield kg ha^−1^	Adjust yield kg ha^−1^	Total variable cost (ETB)	Gross benefit (ETB)	Net benefit (ETB)	MRR%
0	0	1114.8	1003.32	0	7023.24	7023.24	
11.5	10.25 SE	1513.9	1362.51	633.76	9537.57	8903.81	D
11.5	10.25 SE45	1698.05	1528.24	633.76	10697.71	10063.95	479.78
11.5	10.25 S45	1700.15	1530.13	647.76	10710.94	10063.18	D
23	20.5SE	2018.9	1817.01	1085.64	12719.07	11633.43	347.32
23	20.5 SE45	2013.5	1812.15	1085.64	12685.05	11599.41	D
23	20.5 S45	1960.8	1764.72	1099.64	12353.04	11253.4	D
34.5	30.75 SE	2139.9	1925.91	1537.51	13481.37	11943.86	D
34.5	30.75 SE45	2044.4	1839.96	1537.51	12879.72	11342.21	D
34.5	30.75 S45	2476.6	2228.94	1551.51	15602.58	14051.07	518.95
46	41	1928.9	1736.01	1876.94	12152.07	10275.13	D

SE stands for urea application at sowing and at emergence; SE45 stands for urea application at sowing, at emergence and at 45 days after emergence, S45 stands for urea application at sowing and at 45 days after emergence D stands for dominated ETB stands for Ethiopian birr.

**Table 4:** Partial budget analysis for Shumshiha-Lasta Lalibela.

P_2_O_5_ kg ha^−1^	Urea kg ha^−1^	Unadjusted yield kg ha^−1^	Adjust yield kg ha^−1^	Total variable cost (ETB)	Gross benefit (ETB)	Net benefit (ETB)	MRR%
0	0	1023.1	920.79	0.0	6905.93	6905.93	
11.5	10.25 SE	1266.7	1140.03	623.8	8550.23	7926.46	164
11.5	10.25 SE45	1427.3	1284.57	634.3	623.76	9010.51	332
11.5	10.55 S45	1284.6	1156.14	656.8	8671.05	8014.29	D
23	20.5SE	2072.4	1865.16	1075.6	13988.70	12913.06	884
23	20.5 SE45	2807.8	2527.02	1108.6	18952.65	17844.02	14942.3
23	20.5 S45	2045.1	1840.59	1304.4	1075.64	12728.79	D
34.5	30.75 SE	2453.7	2208.33	1527.5	16562.48	15034.96	D
34.5	30.75 SE45	2133.9	1920.51	1527.5	14403.83	12876.31	D
34.5	30.75 S45	1924.4	1731.96	1560.5	12989.70	11429.19	D
46	41	2437.5	2193.75	1883.9	16453.13	14569.18	D

SE stands for urea application at sowing and at emergence; SE45 stands for urea application at sowing, at emergence and at 45 days after emergence, S45 stands for urea application at sowing and at 45 days after emergence D stands for dominated treatment, ETB stands for Ethiopian birr.

## Conclusion and recommendation

Micro-dosing fertilizer application was found very important to increase the production and productivity of sorghum. Results from this study confirm that micro-dosing fertilizer application increases sorghum yield than row application. Hence, at Aybra-Sekota, 30.75 kg N ha^−1^ and 34.5 kg ha^−1^ P_2_O_5_ increased sorghum yield by 28.4% over the recommended rate of 100 kg ha^−1^ DAP and 50 kg ha^−1^ urea whereas, 23 P_2_O_5_ kg ha^−1^ and 20.55 kg ha^−1^ N increased the sorghum yield by 15% over the recommended NP (100 DAP and 50 urea kg ha^−1^) at Shimshiha-Lasta Lalibela areas. Micro-dosing fertilizer application is simple and cheap with low risk to resource-poor farmers in the dry areas of Wag-Lasta. Therefore, the application of 34.5 kg ha^−1^ P_2_O_5_ and 30.75 kg ha^−1^ N (N applied in three splits) for Aybra-Sekota and 23 P_2_O_5_ kg ha^−1^ and 20.5 kg ha^−1^ N (N applied in three splits) for Shumshiha- Lasta Lalibela are recommended for higher sorghum yield and economic utilization of the fertilizers.

### Abbreviations

DAP: Di Ammonium Phosphate, ETB: Ethiopian Birr, RCBD: Randomized completed block design, MRR: Marginal Rate of Return, SAS, Statistical Analysis Software, NP: Nitrogen and Phosphorus.

## Data Availability

We declare that the data used in this manuscript is available if anyone desires to access request the corresponding author.
